# Recovering or working: women’s experiences of working while coping with cancer: a qualitative study

**DOI:** 10.1007/s00520-025-09349-1

**Published:** 2025-03-17

**Authors:** Avital Gershfeld-Litvin, Olga Vishnia, Tsipi Hanalis-Miller

**Affiliations:** 1 School of Behavioral Sciences, The Academic College of Tel Aviv-Yaffo, Rabenu Yeruham 2, 6818211 Tel Aviv-Yaffo, Israel; 2 Sheba Medical Center, Ramat Gan, Israel

**Keywords:** Work, Cancer, Women, Return to work, Rehabilitation

## Abstract

**Purpose:**

The aim of this study was to explore women’s experiences of working and returning to work while coping with cancer.

**Methods:**

Participants were ten Israeli women with cancer who had an active career at the time of diagnosis. Semi-structured interviews were conducted and thematically analyzed.

**Results:**

Four themes were generated. The first was “the meaning of work prior to the diagnosis”: participants shared their perspective on the significance of work in their life prior to being diagnosed with cancer—work was construed as either time-consuming, a source for socializing, or a source for meaning and self-worth. The second was “the diagnosis of cancer and work”: participants held the belief that either the cancer was caused by work or that the cancer halted their careers, in some cases both applied. The third was “the combination of work and cancer treatments”: participants described severe physical, cognitive, and emotional challenges they faced, and how these affected their ability to balance work with receiving treatments. The fourth was “returning to work after cancer”: participants found themselves having to balance preventative and rehabilitative care with career demands, employer expectations, and general work-life balance adjustments.

**Conclusion:**

Findings suggest that healthcare professionals should assess individual perspectives and capabilities prior to returning to work and elucidate opportunities and challenges that cancer survivors may meet. Findings also reaffirm the need for formal workplace education and policies to combat discrimination and tailored return to work opportunities to survivors.

**Implications for cancer survivors:**

Women’s experiences of working while coping with cancer were thematically analyzed. These women face many challenges in the context of returning to work. Findings suggest that returning to work could be facilitated by healthcare professionals and employers through communication and tailored workplace policies.

## Introduction

The survival rate among young adults diagnosed with cancer has increased dramatically in recent decades, with 5‐year survival rates exceeding 80% in most developed countries, including Israel, due to earlier diagnosis and improved treatment [1, 2]. About half of young adults diagnosed with cancer are of working age and can return to work [3]. The ability to return to work (RTW) is a confirmation of the social or family status of cancer survivors and an indication that the cancer has been cured [4]. An important aspect of the quality of life of cancer survivors is labor participation, as work can provide psychological benefits (structure, a sense of normality, and social interaction), as well as practical and economic benefits [4–6]. Despite differences in cancer types and social welfare systems, approximately 49–91% of cancer patients return to work, in a different scope job, within a year worldwide [7–9].

In general, a large proportion of cancer survivors report concern for cancer-related changes to employment [10], as well as difficulties in managing their work due to multiple physical, cognitive, and psychological impairments, and other work-related barriers [11]. About half of all working cancer patients lose their jobs after receiving a cancer diagnosis, report varying difficulties in RTW after treatment, and only up to 40% of them are reemployed [7]. Studies suggest that variations in non-return to work may be attributed to various reasons, yet the key influencing factors appear to be gender, age, income level, medical conditions and comorbidities, the presence or absence of a social support system, the level of understanding from co-workers, the prevailing stigma, and the presence or absence of discrimination toward cancer patients in the workplace [7–9, 12, 13]. Early job loss and delayed re-employment among cancer patients were found to be associated with female gender, younger age, lower level of income, receiving adjuvant treatment, the absence of a support system, and discrimination in the workplace [7, 8]. Early retirement among cancer patients was found to be closely associated with risk factors such as older age, lower level of income, lower level of education, manual labor, sickness leave in the year prior to retirement, and comorbidities [9].

Recent qualitative studies that explored the experiences of cancer survivors upon their RTW found that returning to work was influenced by individual perspectives, the nature of the disease, and access to a support system. Individual cancer survivors held different perspectives (the meaning they ascribed to their RTW and their expectations) that affected their decision to return to work. In this regard, the positive meaning of returning to work as well as consistency between expectations, responsibilities, and physical and emotional readiness, made them eager to return to work. Limited information about the disease and about RTW had made them doubtful about returning to work [6]. Accordingly, four prominent themes could be derived from 28 qualitative studies: (i) Being unable to find meaning after receiving cancer diagnosis; (ii) Concerns and considerations before returning to work; (iii) Reasons for returning to work; and (iv) Life at work after cancer diagnosis and treatment [14].

Insufficient knowledge about cancer and its implications for work was identified as an overarching theme, leading to stigma, misconceptions, and a lack of communication in the workplace, the healthcare system, personal life, and in the broader societal context [15]. According to Bae & Cho (2021), the attitudes of others, especially those of co-workers, affected cancer patients’ working status after diagnosis [7]. Although cancer patients receive the most support from their co-workers, they also felt uncomfortable communicating with them about their condition, as they believed their co-workers were too deeply involved in their daily lives.

Gender, or being a woman, is a variable that was significantly associated with later RTW [16]. Among women, younger age, higher education, high-income, being single, and having positive social support from friends and family, were found to increase the likelihood of RTW [17–20]. In a recent study conducted in Israel, women with breast cancer reported difficulties in RTW, and roughly one third of these women did not return to work [21]. They also expressed their frustration at the lack of rehabilitation services for their condition and needs in Israel. This finding highlights the critical gap in our understanding of women’s experiences with RTW, following cancer, in Israel. Previous research has primarily focused on breast cancer survivors, leaving experiences with other types of cancer largely unexplored.

Israel’s healthcare system provides universal coverage, ensuring that all Israeli citizens receive access to medical services. Participation in a health insurance plan is mandatory and operates under a progressive model. The system functions in accordance with two key laws. First, the National Health Insurance Law of 1995, which requires all citizens to enroll in one of four public nonprofit health maintenance organizations (HMOs). Second, the Patient Rights Law of 1996. The healthcare system fully covers the cost of medical diagnosis and treatment, including both general and preventive care, as well as mental health services and paramedical treatments, such as physical therapy and physiotherapy [22]. Oncological treatments are provided in hospitals and fully covered by the health insurance system. Additionally, cancer patients have access to physiotherapy and physical therapy through the community health services of the HMOs. Moreover, occupational clinics, which operate as parts of the HMO community services, offer support to patients dealing with RTW challenges.

Previous studies in Israel have shown that employers generally demonstrate a positive attitude toward the RTW of cancer patients [22]. However, research on breast cancer survivors in Israel found that approximately 20% of them did not return to work, either due to the lasting effects of cancer and its treatments or due to negative attitudes from employers. Past studies highlight the need to improve the RTW process and its success rate [22]. 

The impact of cancer on employment, particularly on RTW, is a significant element in the quality of life (QoL) of cancer survivors and their families. While existing research has examined various aspects of RTW following cancer, there remains a significant gap in understanding the unique experiences of women with cancer in the Israeli context, where cultural norms, healthcare policies, and workplace practices create distinct challenges and opportunities. Most studies on RTW to date have been quantitative. The few existing qualitative studies on the experiences of women as they relate to RTW focused solely on women recovering from breast cancer. In view of the challenges of RTW, researchers and professionals expressed an urgent need for deeper understanding of RTW perceptions, to enable the development of interventions and an integrative cancer care model based on a holistic biopsychosocial approach [23].

Understanding these experiences may allow for the development and adaptation of tailored interventions that could help women recovering from cancer in dealing with RTW, as well as in the development of interventions by healthcare professionals and employers to support and facilitate employees in their RTW process. Therefore, the aim of the present study was to explore the personal experiences of Israeli women recovering from cancer, the significance of work in their lives, the impact of the diagnosis and treatment on their ability to work, and their decision to return to work after recovery.

## Method

### Study design

This research adopts a qualitative design utilizing semi-structured interviews. This methodology was selected to explore the experiences of women recovering from cancer, particularly regarding their return to work. Semi-structured interviewing is well-established in research for its ability to effectively explore lived experiences and their associated meanings [24]. The study followed the Consolidated Criteria for Reporting Qualitative Research (COREQ) to ensure thorough reporting [25].

### Sample

Participants were recruited through purposive sampling. Advertisements were posted on internet forums and Facebook groups for women cancer survivors with administrator approval. Upon contacting the team via email or phone, candidates were provided with information regarding the study, as soon as their compatibility with the inclusion criteria was ascertained. The inclusion criteria were: (1) Israeli and Hebrew-speaking women; (2) who were diagnosed with cancer and in remission for at least one year prior to their participation in the study; and (3) had an active career and had been employed for at least one year prior to diagnosis.

Ten women, ranging in age from 27 to 57 and meeting the inclusion criteria, were interviewed (see Table 1 for participant characteristics). Before their participation, all women provided written informed consent. The interviews were conducted in Hebrew and spanned from 30 to 90 min, with a mean duration of 60 min. To ensure anonymity, identifying information was excluded from the transcripts, and all study materials were saved under password protection to maintain confidentiality. Quotes were translated into English by the second author, a native speaker of Hebrew with proficiency in English. These translations were approved by the first and third authors, both of whom are native Hebrew speakers with proficiency in English.

**Table 1:** Participant characteristics

Name^a^	Age	Marital Status	Children	Occupation	Diagnosis, Year	Malignancy
Jane	44	Married	2	Teacher	2018	Breast
Abigail	46	Married	1	Advertiser	2012	Breast
Beth	51	Married	None	Coach	2018	Colorectal
Leah	57	In a relationship	1	Teacher	2013	Colorectal
Sarah	44	In a relationship	3	Writer	2017	Colorectal
Mary	27	Married	None	Bookkeeper	2018	Cervical
Sharon	44	In a relationship	None	Artist	2006	Breast
Kate	46	Married	3	Teacher	2015	Breast
Ruth	31	Married	4	Engineer	2015	Lymphoma
Lily	51	Married	3	Teacher	2017	Myeloma

^a^Pseudonyms were assigned to participants to maintain their anonymity

### Data collection

Participants were interviewed by the second author, a female graduate medical psychology student with experience in conducting and analyzing qualitative interviews. The interviews were semi-structured and took place between April and September 2020 (see Table 2 for interview guide). The interviews were conducted on the online video-conferencing platform Zoom, due to social distancing measures set at the time in response to the pandemic.

**Table 2:** The interview guide

1. Please tell me about your career (Where did you work? For how long?)
2. Please tell me about your experience with cancer in relation to your work (Did you continue to work? If so, what was it like to work during the treatment period?)
3. Has work been affected by the cancer diagnosis and its treatments? If so, how?
4. Please tell me about the decision to continue or to stop working
5. If you decided to go back to work, what was it like to go back?
6. What was the significance of work in your life?

A general opening question was used in the beginning of each interview: “Please tell me about your career.” Subsequent questions included follow-up, exploratory, and open-ended questions, such as: “Has your work been affected by the cancer diagnosis and its treatments”; “Please talk about the decision to continue or to stop working”; “If you decided to go back to work, what was it like to go back?”; “What was the significance of work in your life?”.

### Data analysis

Data analysis was performed by all three authors using Braun and Clarke’s method for reflexive thematic analysis [26]. It is a well-established method for analysis of qualitative data. This method consists of six duplicable steps: (1) Becoming familiar with the data; (2) Generating codes; (3) Generating themes; (4) Reviewing themes; (5) Defining themes; and (6) Choosing exemplars. The core of this method is based on the notion that a theme captures a significant aspect of the data in a patterned manner, focusing on generating meaning, rather than scavenging for commonalities or shared elements of the experience [25].

The interviews were transcribed by the second author. Following this stage, all authors reviewed the transcripts to familiarize themselves with the data. Initial codes were generated from each transcript. These codes were then groups into categories owing to a shared conceptual domain. These categories were then synthesized into themes. The authors engaged in deliberations and reflected on their research experience, with the aim of cultivating a nuanced and reflexive interpretation of the data as well as a method of conflict resolution. The outcome comprised of themes and sub-themes, theme descriptions, and exemplars representing each of the sub-themes.

### Researcher reflexivity

The authors acknowledge that our sample size is small, it is important to note, as Braun & Clarke (2019) pointed out, that meaning is not simply uncovered within the data, but rather generated from it, and the interpretation of qualitative data is contextual and subjective [26]. To increase the rigor of the outcomes, some principles of trustworthiness, such as credibility and dependability were considered. The most critical consideration was that of the researchers’ backgrounds, preconceptions, and experiences. We recognize that our experiences inform our research and influence the analysis of the data. Our team has both research and clinical experience with cancer patients and rehabilitation. The authors have experience with qualitative research, particularly in the field of medical psychology. The second author openly shared these basic facts with participants at the start of each interview. Throughout this process, the team reflected on their research experience, engaged in discussions, and provided mutual constructive feedback while reviewing the outcomes of the analysis.

### Ethical approval

The current study received ethical approval from the institutional review board of The Academic College of Tel Aviv-Yaffo, approval number: 2020028.

## Results

Four themes were generated in thematic analysis: “the meaning of work prior to the diagnosis,” “the diagnosis of cancer and work,” “the combination of work and cancer treatments,” and “returning to work after cancer.”

### First theme: “the meaning of work prior to the diagnosis”

The sub-themes of “the meaning of work prior to the diagnosis” were “work as time-consuming,” “work as a source for socializing,” and “work as a source of self-worth and meaning.”

#### Work as time-consuming

Participants reported their jobs required long hours and were highly stressful. Sarah spoke about working under pressure: “I managed the entire company and its projects. I often worked up to 320 h a month under a lot of pressure.” Another participant pointed out her continued investment and constant preoccupation with work: “I will admit that I was quite a workaholic. I was focused on work even outside office hours.” [Abigail].

#### Work as a source for socializing

Several participants described their workplace as a source for connections fostering a sense of belonging. As Lily shared: “I really like meeting people, staff, principals. That's what gives me strength.” Another participant emphasized work as a social connector: “A person needs to work in life. It's also something that connects people. [Ruth].

#### Work as a source of self-worth and meaning in life

Participants described work as essential and fulfilling, often using words like “love” and “pleasure” to describe it. They felt their work was meaningful. Beth shared: “I really, really love my job. I really, really enjoy it.” Another participant spoke about progressing in her career: “I was climbing the ladder at work…I was receiving praise and felt appreciated there.” [Jane]. Other participants described their passion for work. As one participant described: “My work is my way of life. I believe it is my calling.” [Kate].

### Second theme: “the diagnosis of cancer and work”

The sub-themes of “the diagnosis of cancer and work” were “work as a cause of the disease” and “cancer stopped my career.” Participants described a belief that cancer and work were connected in one or two causal relationships—the cancer was caused by work, and the cancer stopped their careers.

#### Work as a cause of the disease

Participants expressed their belief that work pressures are linked to physical illnesses and voiced concerns that future work might trigger a recurrence of their diseases. Kate illustrated this by sharing the following: “Maybe an emotional state led to a mental state and then to a physical state. I used to work with women with breast cancer, maybe I identified too much with them, and that’s why I got the disease.” Another participant, Ruth, shared: “The fatigue from travelling wore me out…This led to a state of exhaustion and the disease took hold. You might disagree, but I feel it was part of it”.

#### Cancer stopped my career

Participants described cancer as a major disruption, striking at the peak of their careers and halting their progress. One participant shared: “I was in a period of great ascent, having just secured a management position.” [Jane]. Leah expressed her frustration, stating: “I was at the peak of my career and had great plans for the future, then cancer came…It stopped and changed my life.” Another participant reflected in sadness: “I was at the height of my success, moving at a crazy pace from one task to another, with a fabulous job.” [Lily].

### Third theme: “the combination of work and cancer treatments”

The sub-themes of “the combination of work and cancer treatments” were “working as a coping mechanism,” “sick at home, healthy at work,” and “focusing on survival.” Participants shared their experiences during cancer treatments, highlighting the severe physical, cognitive, and emotional challenges they faced. They focused on how it affected their ability to work and their attempts to balance work with receiving treatment.

#### Working as a coping mechanism

Amid their cancer struggles, some participants found work to be an anchor and a sign of normalcy. As one participant described: “I quickly returned to school. Staying home, as a cancer patient, felt like declaring myself dead. I continued to work, and scheduled treatments on my days off. It kept me sane.” [Leah]. Another participant echoed this sentiment: “Stopping work completely might have led me to despair. Work was my anchor. Maintaining my routine kept me grounded.” [Kate]. One participant highlighted the social aspect of work, noting that: “Working and meeting people helped me keep my sanity. Interacting with people in a normal way was incredibly helpful.” [Jane].

#### Sick at home, healthy at work

Some participants discussed their efforts to hide their disease at work. As one participant mentioned: “I had a biopsy mark on my neck and big blue bruise, so I always wore my hair in a braid to hide it, somehow people still noticed.” [Ruth]. Another shared: “When I got out, I pretend that everything is fine. I dress up. I put my makeup on. I can’t show anyone that something is wrong, especially at work. It’s not in my nature to complain and be a victim.” [Beth].

#### Focusing on survival

Most participants left their jobs immediately after their diagnosis. As one participant shared: “I left the same day I got the call from the hospital. I collected my things and never returned.” [Ruth]. For those who did not leave their jobs right away the physical and mental toll of cancer treatments made it impossible to work. One participant shared: “All I wanted was to get rid of everything. Work was simply beyond my strength at the time.” [Sharon]. Another participant echoed these sentiments: “I couldn’t even volunteer. I was a broken vessel, half human, sometimes unable to get out of bed.” [Lily]. For some, leaving work was an act of survival: “I felt that if I didn’t leave work, I would collapse.” [Sarah]. Another participant recounted: “I was fighting for my life. You just can’t think about work. You just want to recover.” [Mary].

### Fourth theme: “returning to work after cancer”

The sub-themes of “returning to work after cancer” were “difficulty returning to work due to short and long-term side effects of treatments”, “difficulty returning to work due to negative reactions from employers,” *“*changes in perspective regarding the centrality of work in life,” “cancer as an opportunity to change professional direction,” and “volunteering.” Participants navigated the transition from cancer recovery to resuming work, while focusing on the impact of treatments and their side effects on their abilities. In this process, they found themselves having to balance preventive and rehabilitative care with career demands, employer expectations, and general work-life balance adjustments.

#### Difficulty returning to work due to short and long-term side effects of treatments

Participants reported that the side effects and consequences of cancer and its treatments affected their ability to RTW. For example, Kate spoke about ongoing pain when exposed to extreme temperatures, and persistent nausea: “To this day I suffer from side effects. If it’s hot outside, I’ll feel pain. If it’s extremely cold, I’ll also feel pain. I still get nausea and fatigue.” Another participant shared how she had to adjust her work schedule to accommodate treatments: “I didn’t return to a full-time job. I left one day off for treatments.” [Lily]. One participant found she could not work the same hours: “Even when you go back, you can’t give the same output you gave when you were working before. I used to have no problem exceeding 186 h a month. Now I can’t even reach that.” [Mary].

#### Difficulty returning to work due to negative reactions from employers

Participants noted a shift in employer as well as co-worker attitudes before and after they got sick. One participant recounted with sadness how her boss’s behavior changed due to her reduced capacity: “I used to be considered among the best teachers at the school but suddenly became a burden to my boss…If you are healthy and able to work, they will work you like a donkey; once you get sick, you’re out.” [Jane]. Another participant recalled rejections from workplaces: “There were places where I was told upfront that they would rather not hire someone who could ask for a leave of absence due to health reasons. It breaks you mentally. I did not ask for it at 26 years old.” [Mary].

#### Changes in perspective regarding the centrality of work in life

Participants noted that the disease reshaped their worldviews and work-life priorities. One participant remarked: “Cancer makes you rethink your priorities. I’m not the same person I was before the disease.” [Lily]. Some participants emphasized a need for a renewed, more peaceful balance between work and their personal life. As Sarah shared: “I want to do my job well but keep my family in mind. Nowadays, all I’m looking forward to is some peace.” One participant highlighted a shift in her priorities: “I thought I would work from home from now on. My children come first, then everything else. Before I got sick, I used to focus equally on my career and personal development. That has certainly changed.” [Ruth].

#### Cancer as an opportunity to change professional direction

Some participants mentioned that cancer prompted them to pursue different professional paths they had previously dreamed of but felt they lacked the courage to follow. One participant shared: “I wasn’t satisfied with just being a teacher. I wanted something more. Getting cancer simply sped up a process that would have eventually happened anyway.” [Jane]. Abigail shared a similar sentiment: “I had already had thoughts about starting my own business, but I just lacked the conviction. Cancer gave me the push I need to act.”

#### Volunteering

Several participants felt their personal experiences with cancer engendered a desire to volunteer and help others. One participant used her free time from not working to volunteer: “When I left my job, I had so much free time, I would instead volunteer here and there.” [Sharon]. Another participant even established a non-profit to supports women like her: “Now that I’ve recovered, I focus all my work on volunteering and the organization we founded.” [Lily]. Volunteering gave these women a sense of purpose and meaning. As one participant recounts: “I love being able to give to others…After cancer disrupted my life, giving back is what motivates me daily.” [Beth].

## Discussion

The present study explored the lived experiences of women diagnosed with cancer in relation to their RTW. The findings are encapsulated in four themes that outline the women’s journey, from the initial impact of the diagnosis on their employment through recovery, emphasizing the profound influence of the diagnosis and treatments on these women’s ability and willingness to RTW.

The current findings show that, before their illness, participants expressed varying relationships with work. Women described their careers as central to their identities and daily lives, contributing to self-worth, meaning, and social connections. Participants also described their careers with passion and purpose, referring to them as a “calling” or “way of life”. This supports research showing that RTW is crucial for cancer survivors QoL, offering a sense of meaning, belonging, and reintegration. In contrast, inability to work can lead to loss of confidence and social isolation [27]. The centrality of work in these women’s lives made the disruption and distress caused by cancer especially severe. Some participants held the belief that chronic work stress could explain their illness. While the mind–body link in cancer development is still under study, this perception aligns with findings that chronic stress can facilitate cancer progression through various biological mechanisms [28].

For several women, the cancer diagnosis abruptly halted or ended promising careers. Prior research has highlighted the significant employment disruptions and financial hardships cancer patients face [29, 30]. It is important to recognize that these narratives possibly reflect an idealized version of life before their illness, correspondingly reflecting the perceived magnitude of the life changes engendered by cancer. The present findings illustrate the psychological toll of this career curtailment. While coping with their illness, women showed varying relationships with work. For some, continuing to work, in any capacity, helped them preserve a sense of normalcy, routine, functioning, social connections, and continuity during a tumultuous time. This aligns with past studies showing the benefits of RTW and remaining active for psychological adjustment in cancer patients [16].

Within the Israeli healthcare context, where the system combines public and private services, participants faced unique challenges in balancing their treatment schedules with work commitments. The structure of the healthcare system, including the role of medical committees and disability benefits, influenced their decision-making about continuing work during the treatment phase. Some participants described experiences of concealing their illness at work and making efforts to maintain a professional appearance. These participants reported feeling pressure to separate their “patient” identity from their professional identity. Previous research has documented similar experiences of nondisclosure among cancer patients, often stemming from concerns about workplace stigma and discrimination [15]. Maintaining a strict separation between their “patient” and “professional” identities may have been a strategy to preserve their professional identities and roles. However, this compartmentalization also appears to have taken a heavy emotional toll. Some women felt compelled to immediately leave work and focus solely on survival. Over time, those who initially tried to keep working eventually took sick leave due to the physical, cognitive, and emotional demands of cancer treatment, and ultimately stopped working altogether.

For those who were able to return to work after treatment, significant side effects and long-term impacts made resuming their previous workloads and responsibilities very difficult. Fatigue, cognitive troubles, and pain interfered with their work capacity and performance. Several women felt they were no longer fully qualified to perform up to the standards of their demanding professions. This aligns with extensive literature documenting the high prevalence and persistence of debilitating late effects in cancer survivors in general, and in women recovering from cancer in particular [31, 32]. Additionally, past research revealed distressing levels of cancer-related employment discrimination and failure to accommodate cancer survivors’ new needs [33]. Participants in the present study also reported being viewed as a “burden” to their employer post-illness, and having job offers rescinded due to assumptions about future health problems. The combination of self-doubt about abilities and perceived bias creates formidable barriers to RTW and deserves further study.

Coping with cancer prompted several women to reassess their relationships with work, leading them to seek more work-life balance and less stress. This shift aligns with research indicating that health crises often result in a re-evaluation of life values, fostering posttraumatic growth and transformation [34, 35]. Some participants made intentional choices to find more sustainable positions that accommodated their new realities, while others saw cancer as a wake-up call to pursue entirely new professional paths, including volunteering and entrepreneurship. For those unable to return to their previous jobs, these transitions offered opportunities to channel their experiences into altruistic endeavors and discover new sources of meaning and identity. Past studies have shown that the crisis of cancer can be conducive to positive changes in women’s lives, particularly in their appreciation of life, relationships with others, and self-perception. This implies that these women may become more aware, self-confident, and committed to their loved ones [36].

## Study limitations

While this study provides insights into participants’ experiences, it is important to acknowledge its limitations. The small sample size was considered as a possible limitation; however, the authors subscribe to the notion, supported by evidence across studies, that rigorously collected qualitative data from small samples can substantially represent the full dimensionality of an experience [37]. The retrospective nature of the interviews possibly entails a certain degree of recall bias. Future research could address these aspects by considering a longitudinal design with various points of contact for interviews, to allow for a more in-depth exploration of these experiences.

## Clinical implications

The present study suggests that cancer survivors require more information and support from healthcare professionals regarding RTW, working during treatment, and other employment-related issues. Within the Israeli healthcare system, this support should be integrated into both public and private healthcare services, with clear coordination between medical committees, rehabilitation specialists, and workplace support systems. Employers should prepare the workplace for cancer survivors’ re-entrance as well as necessary support, taking into consideration survivors’ expectations from the workplace [6, 14]. Additionally, the present findings suggest that healthcare providers, including rehabilitation specialists, should assess cancer survivors’ individual perspectives and capabilities, prior to their RTW, and provide them with information about their disease, its treatments, and the opportunities and challenges involved in RTW. The present findings also reaffirm the need for formal workplace education and policy initiatives to combat discrimination, provide accommodating RTW programs, and offer cancer survivors tailored RTW options as they transition between acute treatment and survivorship.

## Conclusions

This study explored the profound impact of cancer on patients’ work lives and identities, highlighting diverse coping and adjustment strategies through career reengagement or reinvention. Ongoing efforts are crucial to alleviate employment burdens for cancer survivors, especially for women, and support their pursuit of work aligned with their new values and priorities. These efforts should be contextualized within Israel’s unique healthcare and employment framework to ensure effective implementation of support systems and policies.

## Data Availability

The datasets generated during and/or analyzed during the current study are available from the corresponding author on reasonable request.
